# Topics of Public Interest

**Published:** 1911-10

**Authors:** 


### TOPICS OF PUBLIC INTEREST

### Marie Feodorovna Prize Competition

**To Be Held in Conjunction with the Ninth International Red Cross Conference, 1912**

**Subjects for Competition**

1. Organization of the methods of evacuation of the wounded on the battlefield, comprising as complete an economy as possible in litter bearers.

2. Portable (surgeons') washstands for war.

3. Methods of packing dressings at the aid stations and in the ambulances.

4. Wheeled stretchers.

5. Carriage of stretcher on mule-back.

6. Folding stretcher easily portable.

7. Transport of the wounded between war vessels, hospital ships, and the coast.

8. The best method of heating railroad cars by a system independent of steam from the locomotive.

9. The best model of a portable Roentgen apparatus, permitting utilisation of $x$-rays on the battle-field and at first aid stations.

**Prizes**

1 First Prize of 6000 roubles (approximately $3,000).

2 Second Prizes of 3000 roubles (approximately $1,500) each.

6 Third Prizes of 1000 roubles (approximately $500) each.

**When and Where to be Awarded**

Inventions entered in this competition are to be displayed at an exhibition to be held on the occasion of the Ninth International Red Cross Conference at Washington, D. C., May 7-17, 1912.

All persons intending to compete for these prizes must forward to the Chairman of the Exhibition Committee, at the above address on or before December 31, 1911, a statement of such intention, giving the number of cubic feet which will be required for the exhibition of their inventions.

Articles entered in this competition must be received, carriage prepaid, at Washington, D. C., on or before April 15, 1912

Further information may be obtained from the Chairman of the Exhibition Committee.

### Trained Nurse—Philippine Service

THE United States Civil Service Commission invites particular attention to the need of trained nurses in the Philippine Service. This Commission to which application should be made in advance, has announced an examination to be held on October 18. This service is an excellent field for graduate nurses, and offers many opportunities and advantages not found in other parts of the Government service. The hours of service are short and vacation leave affords an opportunity for travel in the Islands, China, and Japan. The age limits are 20 and 40.

More than three-fourths of the nurses are on duty at the Philippine General Hospital, Manila, which is a modern institution of 350 beds, for the treatment of acute diseases.

The contract period is two years, and the salary is $50 per month for the first six months, with subsistence, quarters, and laundry. This is a probational period, and the appointment will not be made permanent unless the nurse's work has been satisfactory. If her services are satisfactory, at the end of the six months she is given a regular appointment at $60 per month, with subsistence, quarters, and laundry.

---

CONTRACT PRACTICE. The Committee of the Medical Society of the County of Erie held a meeting on September 18th. The Chairman, Dr. Edward E. Haley, summarised the results as follows: 435 replies have been received from members agreeing to abstain from contract practice, according to the definition formulated. About five refusals have been received. About 100 members have either not been heard from or have replied unfavorably. However, so far as the committee could judge, more than half of this number have merely neglected to reply or have been out of town and are really favorable to the recommendations of the committee. Some of the unfavorable replies seem to be due to unwillingness to commit one's self rather than to practice on contracts and some are due to a misunderstanding of the exact definition adopted. Much amusement was caused by a profane and badly spelled anonymous postal card sent to Dr. Haley by an advocate of contract practice. Quite a number of letters were received from distant parts, commending the work of the Committee and it was learned that Norristown, Pa., Bucks Co., Pa., Niagara Falls, Lockport, Jamestown, N. Y., already have in force agreements similar to that proposed for Erie Co. It was also reported that every Italian physician in the city, whether a member of the County Society or not, had pledged himself against contract practice. This is an important matter since this race is particularly prone to benevolent organisations and has resorted largely to medical attendance on a contract basis, without regard to the extent of service required. According to the latest information, about 33 physicians in Buffalo are doing contract work.

---

Economics of Tuberculosis. All are familiar with the arguments based on the valuation of human lives. Such arguments seem to us fallacious. In the first place, they tend to distract attention from strictly humanitarian motives. In the second place, granted a reasonably well populated district, it is questionable whether a human life really has a money value, except when the death of the supporter of a family throws the dependents upon the charity of the community. With very rare exceptions, there are many more persons capable of filling a position than there are chances of employment in that particular position. Dr. J. Ed Dube (*Le Journal de Medecine et de Chirurgie,* Montreal, September 1911) makes a more convincing economic argument for the prevention of tuberculosis. He estimates that of 5,000 consumptives at Montreal, 1,000 require free care in some sort of institution, for an average of a year each—not necessarily at the same institution or continuously—at an average cost of $1.20 a day—total $438,000. Placed in proper sanitariums, the cost would be only $1.00 a day and 700 would be cured at the end of 250 days on the average—$175,000. The 300 fatal cases, would cost for an average of a year, $109,000. Total $284,000, saving $154,000, besides the economic value of the persons restored to health.

Nathan Straus of New York has been appointed by President Taft to represent the United States at the International Congress for the Protection of Infants, in Berlin, September 11-15, 1911; also at a congress to consider the problem of tuberculosis at Rome, to be held next spring. Nathan Straus has probably saved more lives than can be credited to the direct ministrations of any physician and he has done so by following expert advice and co-operating with professional philanthropy. His appointments are at once a recognition of his past services and an opportunity for further usefulness. May we add an appreciation of the breadth of his philanthropy in ignoring race lines and a protest against the stale joke as to the reputed lack of generosity of his race? So far as our experience goes, no people are more ready to respond, according to their ability to a charitable appeal, none less limited by racial or religious prejudice—with one important exception, that they are too proud to let the burden of their own poor fall on others.

---

Mrs. E. H. Harriman has given $50,000 to Dr. F. K. Ainsworth, Chief Surgeon of the Southern Pacific R. R. system, to establish a bacteriologic and pathologic laboratory for the benefit of the employes of that system. It is expected that an expert will be obtained from the Rockefeller Institute.

---

At the International Hygiene Exhibition at Dresden a department is devoted to showing how fear of sickness in days of old gave the quack and the charlatan a large field for the sale of what they called "preventives." The figure of a woman "guarded against cholera," has a wool abdominal band, a bodice of thick elastic material, a belt adorned with small porous bricks and fastened with a waxed silk ribbon. In the ample sleeves there are tiny brushes and little bags of sand to catch and absorb the "poison," while little tubes containing vinegar and boxes of chalk are concealed under high hair puffs. The top of the hat is provided with a "pinwheel," which revolves when the wearer walks and "keeps the air pure about the wearer."—*Tribune.*

---

Sterilised Solutions for Hypodermic Use.—In view of the pronounced demand for sterile "ready-to-use" solutions of definite dosage, to be administered hypodermically, Parke, Davis & Co. some time ago decided to place a number of such solutions at the disposal of the profession in a form that would make their use both convenient and economical. "Sterilised Solutions in Glaseptic Ampoules" is the term used to designate them, and the company announces about a dozen preparations which it is prepared to supply.

The sealed glass ampoule removes the liability of contamination and deterioration, and eliminates the inconvenience attaching to the preparation of a solution whenever an emergency calls for its use. Moreover, it insures medicaments of established purity and strength. Each package contains a small file by means of which the neck of the ampoule is nicked, so that it may be readily broken off, thus opening the container. An ordinary hypodermic syringe is used. To withdraw the liquid, the needle is inserted to a point about midway of the sloping shoulder while the ampoule is held in a vertical position; by this means the solution is removable, we are told, to the "last drop."

Our readers are advised to consult the display announcement of these sterilised solutions appearing in the advertising pages of this issue of Buffalo Medical Journal, which gives a complete list of the preparations as well as some important suggestions for their use.

